# Influence of the Growth and Development Check (GDC) on Overweight/Obesity of Children under-5 Years in China: A Propensity Score Analysis

**DOI:** 10.3390/ijerph18031203

**Published:** 2021-01-29

**Authors:** Chongli Duan, Li Mei, Tingshuai Ge, Quanbao Jiang

**Affiliations:** 1 School of Public Policy and Administration, Xi’an Jiaotong University, Xi’an 710049, China; dcl0121@stu.xjtu.edu.cn (C.D.); meili1246@stu.xjtu.edu.cn (L.M.); 2 Institute for Population and Development Studies, Xi’an Jiaotong University, Xi’an 710049, China; qbjiang@xjtu.edu.cn

**Keywords:** children, China, growth and development check, overweight/obesity, PSM

## Abstract

To improve health and reduce the rapidly increasing prevalence of overweight and obesity in children, the Chinese government has formulated childcare standards, with the Growth and Development Check (GDC) as the main content. However, few studies have evaluated the impact of the GDC on lowering the risk of childhood overweight and obesity. Using the 2014 China Family Dynamics Survey and propensity score matching (PSM), this article examined the impact of the GDC on overweight/obesity in children aged 5 years and younger. The results revealed that the mean Body Mass Index (BMI) was 17.80 kg/m^2^, and the prevalence of overweight/obesity was 24.62% in children. Children whose parents were aware of the GDC had a lower BMI and a lower risk of overweight/obesity than those whose parents were not aware of it. Children who engaged in the GDC in the last 12 months had a lower BMI and a lower chance of overweight/obesity than those not engaged.

## 1. Introduction

Childhood overweight/obesity has become one of the biggest public health issues in the 21st century. The prevalence of overweight/obesity in children has grown globally since 1970, and has increasingly become the most serious health concern for children. According to the data from Worldwide Health Organization (WHO) [1], the number of overweight/obese children aged 0–5 in the world increased from 32 million to 41 million from 1990 to 2016, and is projected to exceed 70 million in 2025. The prevalence of underweight and stunted growth in Chinese children has decreased dramatically with rapid socioeconomic progress and better living conditions, but the prevalence of overweight/obesity has increased rapidly. The Third National Survey on Childhood Obesity in China in 2006 showed that the prevalence of overweight and obesity among children under the age of 6 was 7.2% and 19.8%, respectively. While the prevalence of overweight/obesity in children has steadily stabilized and even started to decrease in some developing countries (e.g., United States, England, France, and Australia) [2,3,4,5,6], the prevalence of overweight/obesity among Chinese children is still increasing rapidly, and is predicted to surpass that of developed countries [7,8]. Childhood overweight/obesity will not only lead to metabolic syndrome, type 2 diabetes, and cardiovascular disease, but also proceed into adulthood, bringing serious economic burdens to individuals, families, and society [9].

The Growth and Development Check (GDC), referring to the monitoring and evaluation of the growth and development status of children aged 0–6, is the main content of China’s child health care. Since 2009, the Chinese government successively formulated the “National Standards for Child Health Care”, the “National Standards for Basic Public Health Services”, and the “Children’s Development Program in China”, which sets particular criteria for the content, frequency, implementation, and assessment indicators of GDC [10,11,12,13,14]. The primary content of the GDC includes the examination of height, weight, and head circumference, excluding disease treatment examinations [15]. As regards the frequency of the GDC, children aged 0–1 years old should have it at least four times, children between the ages of 2 and 3 should have it at least two times, and children above the age of 3 should have it at least one time per year [16]. Thus, the anomalies in the development phase of children can be found in time by conducting the GDC, which may contribute to lowering the incidence of children’s overweight and obesity. Additionally, as one of the basic public health services, the Chinese government requires that the GDC be implemented by health personnel in township health centers, community health service centers, and village clinics. To ensure the smooth implementation and a high participation rate of the GDC program, the Chinese government requires that the GDC participation rate of children aged 0–6 reach over 80% [14,17]. The results of the assessment will be linked to the unit’s funding subsidies and the appointment or dismissal of the main leaders, and serve as the basis for personnel rewards, punishments and wages [18].

To ensure that children’s parents recognize the importance of the GDC, the Chinese government has also put forward specific requirements for the publicity of the GDC at the grassroots level. The Chinese government pointed out that it is necessary to carry out the publicity of the GDC, as well as health education on healthy lifestyles, such as reasonable diet and weight control for parents of children aged 0–6 [12,13]. In terms of service form, primary health personnel are required to provide promotional materials to children’s parents, set up bulletins, conduct public health consultation activities, hold health knowledge lectures, and carry out individual publicized program [12,13]. Besides this, many assessment indicators have been set by the Chinese government to ensure the publicity effect of the GDC. These assessment indicators include the following: the types and quantities of printed materials issued; the types of audiovisual materials broadcast, frequency and time; the publicity column setting and the update of publicity content; the number of lectures and consultation activities, and the number of participants [12,13]. The assessment results of the publicity of the GDC will also be related to the promotion of unit leaders and the rewards and punishments of primary health personnel.

However, the level of GDC awareness and engagement is still very limited. The 2013 National Health Literacy Survey of Urban and Rural Residents showed that only 9.48% of Chinese residents have health literacy in basic health awareness and principles, health skills, and healthy lifestyles and behaviors [19]. Poor health literacy and grassroots political organizations’ neglect of publicity for GDC has left many parents ignorant of the importance of children carrying out regular GDC. Only 41~58% of parents in multiple surveys are aware of the significance of GDC [20,21,22]. This has also, to a certain degree, contributed to a lower level of GDC engagement in Chinese children. In a survey on the GDC engagement of children under the age of 5, Zhao et al. [15] found that only 59.3% of children engaged in regular GDC, with an annual average of 1.4 times; the percentages of children aged 1 year and below, 1–2 years old, and 3 years and older who met the GDC frequency requirement specified in the “National Children’s Health Work Regulations” were only 19.4%, 33.9%, and 53.1%, respectively.

Moreover, there are only a few studies that have evaluated the impact of GDC on childhood overweight/obesity. Using the physical examination data of 220 children aged 3 to 6 years old in the Maternal and Child Health Hospital, Wu et al. [23] found that testing children’s body mass index (BMI) every 4 months can significantly reduce the risk of overweight and obesity in children. Using the data of 188 children in other Maternal and Child Health Hospitals, Huang et al. [24] also found that the regular monitoring of BMI can keep children’s BMI within a reasonable range, and significantly reduce the risk of children’s overweight and obesity. Using the physical examination data of 2000 children aged 0–36 months at the community health service center, Ge [25] found that regular physical examinations can reduce the prevalence of low weight, wasting, growth retardation, and overweight/obesity. Other studies using children’s data in communities have also found this conclusion [26,27]. Although no empirical evidence was provided, some studies conducted in western countries recommended that monitoring children’s BMI is an effective approach to reduce the risk of obesity [28]. Only the US Preventive Services Task Force (USPSTF) found evidence that screening and behavioral interventions for obesity in children 6 years and older can contribute to the improvements in weight status, and the magnitude of this benefit is greater than with pharmacotherapy interventions [29].

Several shortcomings in previous studies were revealed after a closer look at the literature. Firstly, small samples (*N* < 500) from hospitals or health centers were often used in previous research, resulting in a lack of representative samples and a certain degree of selectivity. Secondly, prior studies mostly used descriptive analyses and conventional statistical methods, which did not control for the effect on the association between GDC and childhood overweight/obesity of other confounding variables. Finally, although a control group and an experimental group were formed in some studies to investigate the impact of GDC on overweight/obesity in children, the experimental environment was not strictly controlled. Thus, there are still significant variations between the experimental group and the control group within certain demographic characteristics, which will affect the reliability of the results. Therefore, this study aimed to examine the impact of GDC on the risk of childhood overweight/obesity using the 2014 China Family Dynamics Survey (CFDS) and propensity score matching (PSM). Specifically, this study has the following objectives: (a) investigating the influence of awareness of the GDC on the risk of childhood overweight/obesity to examine the importance of GDC publicity; (b) examining the effect of engagement with the GDC on the risk of childhood overweight/obesity, to assess the actual effect of the GDC program. Below, the data and method are first presented, then the results, and finally the discussion and conclusions.

## 2. Data and Method

### 2.1. Data

The data used in this study were from the China Family Dynamics Survey (CFDS). The National Health and Family Planning Commission (renamed “National Health Commission” after 2018) has conducted the CFDS every two years since 2014 to comprehensively and consistently understand the socioeconomic status of different families, especially family planning families. The 2014 baseline survey covered 30,000 households in 300 counties (districts, cities) of 31 provinces through a multi-stage PPS (probability proportionate to size sampling). In 2016 was the first round of investigations for follow-up. The survey adopted a face-to-face survey method, and all the survey data were collected using the computer-assisted personal interviewing (CAPI) system. The data collection was approved by the Ethics Committee of National Health and Family Planning Commission. Each participant was informed of the purpose of this survey and was voluntary, and were told their privacy would be strictly protected.

This study used the 2014 CFDS because the information on GDC (Growth and Development Check) was only inquired after in the baseline surveys. Since we only focused on the overweight/obesity risk of children aged 0–5, we excluded the information of people aged 5 above, which left an analytical sample of 7149 children aged 0–5. Besides this, since children’s birthweight data were absent from the 2014 CFDS, we imported the birthweight data from the 2016 CFDS, resulting in a reduction in the sample size to 4520. After excluding children with missing values in the variables of concern, the final analysis sample included 3067 children aged 0–5. Among them, 54.03% were boys and 45.97% were girls; 29.44% of the children lived in urban areas, and 70.56% of the children lived in rural areas.

### 2.2. Measurements

Our dependent variables were children’s BMI (body mass index) and overweight/obesity. BMI was defined as weight in kilograms divided by the height in meters squared (kg/m^2^). The height and weight of children were reported by the children’s parents. Unlike adults who can directly determine whether they are overweight/obese based on the range of BMI, for children we need to calculate the body mass index z-scores (BMIz). BMIz scores, also called the BMI standard deviation scores, are measures of relative weight adjusted for a child’s age and sex. Given a child’s age, sex, BMI, and an appropriate reference standard, a BMIz can be determined [30]. The calculation of BMIz is detailed in other studies [31,32]. In line with previous studies [33,34], overweight/obesity was defined as BMIz >2, while non-overweight was defined as BMIz ≤2.

The independent variables in this study include awareness of GDC and engagement with GDC. “Are you aware that the child needs to have regular GDC?” was used to measure the awareness of GDC, with 0 = unaware, 1 = aware. The engagement with GDC was measured by the question “How many times has the child engaged in GDC in the last 12 months?”, with 0 = non-engaged, 1 = engaged in at least one time.

Covariates include variables that represent children’s and their parents’ socio-demographic characteristics, and family economic status. Children’s characteristics include birthweight (0 = no, 1 = macrosomia), mode of delivery (0 = no, 1 = cesarean delivery), gender (0 = female, 1 = male), place of residence (0 = rural, 1 = urban), age, and left-behind status. For left-behind status, children who were left at home because both parents or one of their parents worked outside for more than 6 months each year were regarded as left-behind, and their left-behind status was coded as 1 and otherwise was coded as 0. Parental characteristics include maternal education (0 = no, 1 = above high school), maternal job (1 = agricultural, 2 = non-agricultural, 3 = other), paternal education (0 = no, 1 = above high school), paternal job (1 = agricultural, 2 = non-agricultural, 3 = other). Family economic status was measured using household income (yuan, in logarithm) in the last year. The definitions and measurements of the variables used in this study are shown in Table 1.

### 2.3. Method

Propensity score matching (PSM) was used to investigate the impact of GDC on children’s weight status. Simply incorporating the confounding variables into the regression model may cause collinearity due to the correlation between confounding variables and independent variables. In the current research, for example, differences in parents’ education, occupational status, and family economic status that may lead to the awareness of GDC are not random, but are rather selective. To eradicate selectivity bias and extract the causal estimates between the independent variable and the dependent variable, PSM can match treatment group and control group individuals with the same or identical propensity scores (referring to the conditional probability that the individual understudy is affected by a certain independent variable under the control of observable confounding variables) [35]. PSM has the following advantages relative to conventional statistical methods [35,36]: (a) PSM is a non-parametric model and is not constrained by the setting method in the traditional linear model; (b) it can ensure the comparability of the treatment group and the control group, and those with too high or too small a propensity score will be excluded in the final analysis; (c) PSM is more statistically efficient because of the fewer estimated coefficients.

According to the previous studies [35,37,38], nearest neighbor matching, Caliper matching, and Kernel matching are the most used matching methods in PSM. Among them, nearest neighbor matching refers to matching two groups of individuals when the absolute value of the discrepancy between the estimated propensity score in the treatment group and that in the control group is the smallest. Caliper matching refers to matching two groups of individuals when the absolute value of the discrepancy between the estimated propensity score in the treatment group and that in the control group is within a certain preset range. Kernel matching is more complex compared with nearest neighbor matching and Caliper matching. It takes the weighted average of all individual outcome variables in the control group as the matching value of the individual outcome variables in the treatment group.

### 2.4. Analytic Strategy

We first conducted the descriptive statistical analysis of the full sample and the sub-samples separated according to the awareness of and engagement with GDC. Secondly, OLS (ordinary least squares) and binary logistic regression were used to investigate the relationship between the awareness of and engagement with GDC with children’s BMI and overweight/obesity. Finally, PSM was used to examine whether there is a causal correlation between the awareness of or engagement with GDC and children’s BMI and overweight/obesity. Three different matching methods, including nearest neighbor matching, Caliper matching, and Kernel matching, were performed respectively in PSM to check for the consistency of findings. Referring to previous studies, the preset range in Caliper matching was within one-quarter of the standard deviation of the estimated propensity; the default bandwidth was used for Kernel matching [39,40]. Results in this study were considered significant if *p* < 0.05. All statistical analyses were performed using the *psmatch2* command in Stata^TM^ Version 13.1 software (StataCorp, College Station, TX, US).

## 3. Results

### 3.1. Descriptive Results

Table 2 displays the descriptive results of the variables in the sample. The mean BMI of children aged 0–5 was 17.80 kg/m^2^ and the prevalence of overweight/obesity was 24.62%. In total, 73.98% of children had parents who were aware of the necessity of GDC, and 64.17% of children had engaged in at least one GDC in the past 12 months. Children whose parents were aware of the GDC had a lower BMI (17.57 kg/m^2^ vs. 18.44 kg/m^2^) and a lower risk of being overweight/obese (21.38% vs. 33.83%) than those whose parents were not aware of GDC. Children who had engaged in the GDC in the last 12 months had a lower BMI (17.55 kg/m^2^ vs. 18.23 kg/m^2^) and a lower risk of being overweight/obese (20.78% vs. 31.48%) than those not engaged. In terms of the covariates, the results in Table 2 indicate that children born by cesarean section, living in urban areas, were not left-behind, and with higher family socioeconomic status (parents with higher education, professional prestige, and income) are more likely to have engaged in the GDC in the past 12 months, and their parents are more likely to be aware of the GDC.

### 3.2. OLS/Binary Logistic Regression Results

Table 3 shows the results of the OLS (ordinary least squares) and binary logistic regression for the effect of GDC on children’s weight status. Model 1 and Model 2 show the OLS regression results for the influence of the awareness of GDC and the engagement with GDC over children’s BMI, respectively; Model 3 and Model 4 are the binary logistic regression results for the impact of the awareness of GDC and the engagement with it on the children’s overweight/obesity risk, respectively. The results of Model 1 and Model 3 suggested that children whose parents were aware of the GDC had a lower BMI (*β* = −0.658, *p* < 0.001) and a lower risk of being overweight/obese (*β* = −0.434, *p* < 0.001) than those whose parents were not aware. The results of Model 2 and Model 4 indicate that children who had engaged in the GDC in the last 12 months had a lower BMI (*β* = −0.492, *p* < 0.001) and a lower risk of being overweight/obese (*β* = −0.380, *p* < 0.001) than those not engaged.

### 3.3. PSM Results

Table 4 shows the PSM results of the influence of GDC on children’s BMI and overweight/obesity, which were consistent with the results of OLS and binary logistic regression. Taking the results of Caliper matching as an example, children whose parents were aware of the GDC had a lower BMI (Average Treatment Effect on the Treated (ATT) = −0.669, *p* < 0.001) and a lower risk of being overweight/obese (ATT = −0.103, *p* < 0.001) than those whose parents were not aware. Children who had engaged in at least one GDC in the last 12 months had a lower BMI (ATT = −0.344, *p* < 0.05) and a lower risk of being overweight/obese (ATT = −0.062, *p* < 0.001) than those not engaged. The estimated results of three matching methods were consistent, suggesting that the relationship between the awareness of or the engagement in GDC and children’s BMI and overweight/obesity was very robust.

## 4. Discussion

Using the 2014 China Family Dynamics Survey (CFDS) and propensity score matching (PSM), we examined the influence of the awareness of and engagement in the Growth and Development Check (GDC) on BMI and the risk of being overweight/obese in children aged 5 years old and younger. The results showed that the mean BMI of children was 17.80 kg/m^2^, and the prevalence of overweight/obesity was 24.62%. Children whose parents were aware of the GDC or who had engaged in at least one GDC in the last 12 months had a lower BMI and a lower risk of overweight/obesity than those whose parents were not aware of it or those who were not engaged in it.

Of the 3067 children, 73.98% of the children’s parents were aware of the necessity of regular GDC, and 64.17% of the children had engaged in at least one GDC in the last 12 months. The proportion of parents in the current study who were aware of the GDC is higher than the 41~58% reported in previous studies [20,21,22]. This may be attributed to the promotion by the government of the value of health care for children and the improvement of people’s health awareness. However, although 73.98% of parents know that children should receive regular GDC, only 64.17% of children engaged in GDC in the past 12 months. Additionally, the awareness of and engagement in GDC still vary greatly among families of different economic status. In the current sample, parents living in rural areas, with a lower education level, occupational prestige and income, were less likely to know about GDC or have their children engaged in GDC in the last 12 months, which is consistent with the earlier studies [21,41].

Being aware of the GDC for parents was associated with a lower risk of overweight/obesity in children. We found that children whose parents were aware of GDC had a lower BMI and a lower risk of being overweight/obese. Parents who were aware of the GDC in this study were those with a higher education level, occupational prestige, and income, and thus had a certain reserve of child health knowledge or had more opportunities to learn about child health knowledge. In contrast, parents with a lower socioeconomic status often disregard GDC’s significance, stick to the principle that “a child is healthy if they are not sick”, [15], are relatively inactive in acquiring child health knowledge [41], and also tend not to educate their children in the correct eating habits. For example, among the 37,020 children aged 0–6 years in the rural area of Changchun city (a city in Northeast China), 60% of the children have unhealthy eating habits [42]; in a survey in rural areas of Sichuan Province, Li et al. [43] found that the proportions of unhealthy eating habits, such as picky eaters and excessive snacking, among children aged 0–3 and 4–6 were 51.03% and 79.92%, respectively. Therefore, the promotion of GDC, and especially the promotion for families with lower socioeconomic status, may contribute to increasing parents’ knowledge about childcare and improving their awareness of reasonable diet and weight control. Parents will therefore pay more attention to providing children with reasonable meals, helping children develop good eating habits, which may help reduce the risk of overweight and obesity in children.

The engagement with GDC was associated with a lower risk of overweight/obesity in children as well. We found that children who engaged in at least one GDC in the last 12 months had a lower BMI and a lower risk of being overweight/obese, which is consistent with several previous studies [23,24,25,26,27]. During the process of GDC, child health professionals will guide parents towards childcare based on a comprehensive evaluation of the child’s growth and development and the government’s recommendations [44]. Therefore, through the GDC, children’s abnormalities and diseases in the growth process can be detected in time, and effective interventions can be carried out to correct parents’ misconceptions and improper methods (e.g., neglecting to provide children with reasonable meals and condoning children’s bad eating habits) in childcare, which may contribute to reducing the incidence of overweight and obesity in children [16,17,44]. However, we found that only 64.17% of children in the current sample had engaged in GDC in the past 12 months, which is lower than the 80% GDC participation rate required by the government. The reason may be that the socioeconomic conditions of different regions in China are quite different, and some poor and remote areas struggle to meet the conditions for implementing a regular GDC program [45,46]. Rapid population mobility and migration also make it difficult for migrant children to receive basic public health services, including the GDC program [47,48].

It is worth noting that in addition to requiring children to engage in GDC, improving parents’ awareness of GDC by publicizing it may be another effective way to reduce the risk of childhood overweight/obesity. We found that children who engaged in the GDC were 6.01% (1 − $e^{-0.062}$, the result of Caliper matching in Table 4) less likely to be overweight/obese than those not engaged. By contrast, children whose parents were aware of the GDC were 9.79% (1 − $e^{-0.103}$, the result of Caliper matching in Table 4) less likely to be overweight/obese than those whose parents were not aware. The Chinese government requires primary health personnel to promote the GDC program, and other basic health knowledge and skills for parents of children aged 0–6, using various methods [12,13]. The content of these propagandas can help children’s parents pay attention to the care of children in their daily lives, and prevent children from living in an obese environment. In contrast, GDC is carried out regularly. In the time between the two intervals, it is still necessary for the parents to reasonably control the child’s weight status based on the existing knowledge of childcare and the recommendations from child health professionals during the last GDC. Besides this, children’s GDC participation rate could be improved if parents understand the importance of GDC. For example, Yang et al. [49] found that the distribution of brochures, telephone consultations, lectures, and other forms of education about childcare for parents can significantly increase children’s participation rate in GDC. Other studies have also reached this conclusion [50,51,52]. Therefore, as another potential approach to reducing the risk of overweight and obesity in children, with less cost but a greater effect, primary health personnel should pay more attention to the extensive publicity of the GDC program.

There are some limitations and strengths in this paper. As regards the limitations, first, the children’s height and weight used in this study were reported by their parents. The self-report data may deviate from the actual situation, compared with the data assessed by trained investigators. Secondly, there were no available methods or software packages for us to compute statistical power and sample size for PSM. However, based on the rules of sample size selection [53], and the related research [54] that pointed out that even in the case of small study samples or a low prevalence of treatment, propensity score matching can yield an unbiased estimations of treatment effect, the model used in this study had high statistical power and the sample size was adequate. Lastly, PSM can only calculate the propensity score based on the observed variables, and cannot control the unobserved variables. Although the GDC program is near-mandatory in actual execution, there is still a certain degree of personal choice motivation in GDC participation, which means PSM may not be able to obtain the net effect of GDC on the risk of childhood overweight/obesity. Nevertheless, to our best knowledge, this study is the first study to use national survey data and causal inference methods to investigate the impact of GDC on children’s overweight/obesity. This study has the following strengths relative to previous studies: first, this study used the PSM to analyze the role of GDC in reducing the risk of obesity/overweight. PSM will remove the selection bias caused by confounding variables in contrast with conventional statistical methods to some extent, thereby obtaining the impact of GDC on childhood overweight/obesity. Secondly, this study used nationwide survey data, which had greater representativeness compared to prior research using the data from a certain metropolitan region, community, or hospital.

## 5. Conclusions

The findings of this study indicate that Growth and Development Checks (GDC), including the awareness of and engagement in GDC, would contribute to reducing the risk of overweight/obesity in children. Considering the importance of GDC in lowering the risk of childhood overweight and obesity, China has incorporated it into the national basic public service norms, and put forward the objectives, content, responsible subjects, and assessment indicators of GDC in the “National Child Health Work Standards” and “National Basic Public Health Service Standards”. However, there are still many problems regarding the actual implementation of GDC. For example, the actual participation rate of GDC in this study only reached 64.17%, which is lower than the 80% required by the Chinese government. In addition to the lack of conditions for implementing the GDC program in some remote poor areas and the rapid population mobility and migration between regions, the failure of primary health personnel to enforce the policies as required could cause the low participation rate in GDC as well. Moreover, much attention should be paid to the monitoring of the BMI of children with lower socioeconomic status in families, who are at high risk of obesity. Finally, the promotion of GDC is also of great significance for contributing to an increase in the participation rate of GDC and reducing the risk of childhood obesity. Therefore, the government should increase funding for child health care, especially for children living in low socioeconomic regions or families, and attach priority to the construction of primary health personnel. Primary health personnel should also change their traditional concepts and take the initiative to provide healthcare services, adopt new publicity methods such as distributing brochures and performing telephone consultations, and conduct community lectures to raise the awareness and participation rate of GDC.

## Figures and Tables

**Table 1:** Definitions and measurements of variables.

Variables	Definitions and Measurements
Dependent variables	
BMI	child’s weight in kilograms divided by their height in meters squared (kg/m^2^)
Overweight/obesity	whether the child was overweight/obese; 0 = non-overweight, 1 = overweight/obesity
Independent variables	
Aware of GDC	whether the child’s parents were aware that children need to have regular GDC; 0 = unaware, 1 = aware
Engaged in GDC	whether the child engaged in GDC in the last 12 months; 0 = non-engaged, 1 = engaged
Covariates	
Birthweight	child’s birthweight; 0 = no, 1 = macrosomia
Mode of delivery	child’s birth mode; 0 = no, 1 = cesarean delivery
Gender	child’s gender; 0 = female, 1 = male
Place of residence	child’s resident place; 0 = rural, 1 = urban
Age	child’s age; age in month
Left-behind status	child’s left-behind status; 0 = no, 1 = left-behind
Maternal education	education level of the child’s mother; 0 = no, 1 = above high school
Maternal job	job of the child’s mother; 1 = agricultural, 2 = non-agricultural, 3 = other
Paternal education	education level of the child’s father; 0 = no, 1 = above high school
Paternal job	job of the child’s father; 1 = agricultural, 2 = non-agricultural, 3 = other
Household income	household income of the child in 2013 (yuan, in logarithm)

Note. GDC= Growth and Development Check.

**Table 2:** Descriptive results of variables in the sample.

Variables	Full Sample (*N* = 3067)	Aware (*N* = 2269)	Unaware (*N* = 798)	Engaged (*N* = 1968)	Non-Engaged (*N* = 1099)
BMI			***		***
Mean	17.80	17.57	18.44	17.55	18.23
SD	3.34	3.15	3.74	3.18	3.57
Overweight/obesity			***		***
Overweight/obesity	24.62	21.38	33.83	20.78	31.48
Non-overweight	75.38	78.62	66.17	79.22	68.52
Birthweight					
Macrosomia	7.43	7.80	6.39	7.83	6.73
No	92.57	92.20	93.61	92.17	93.27
Mode of delivery			***		***
Cesarean	44.67	47.29	37.22	48.42	37.94
No	55.33	52.71	62.78	51.58	62.06
Gender					
Male	54.03	54.65	52.26	55.23	51.87
Female	45.97	45.35	47.74	44.77	48.13
Place of residence			***		***
Urban	29.44	34.46	15.16	36.59	16.65
Rural	70.56	65.54	84.84	63.41	83.35
Age			***		***
Mean	24.86	23.99	27.35	23.46	27.38
SD	14.03	14.03	13.75	13.96	13.83
Left-behind status			***		***
Left-behind	20.67	16.88	31.45	16.82	27.57
No	79.33	83.12	68.55	83.18	72.43
Maternal education			***		***
Above high school	23.31	28.87	7.52	30.28	10.83
No	76.69	71.13	92.48	69.72	89.17
Maternal job			***		***
Agricultural	26.93	23.49	36.72	21.75	36.21
Non-agricultural	52.17	55.71	42.11	57.72	42.22
Other	20.90	20.80	21.18	20.53	21.57
Paternal education			***		***
Above high school	24.00	29.75	7.64	31.50	10.56
No	76.00	70.25	92.36	68.50	89.44
Paternal job			***		***
Agricultural	20.80	18.69	26.82	17.58	26.57
Non-agricultural	77.70	79.68	72.06	80.79	72.16
Other	1.50	1.63	1.13	1.63	1.27
Household income			***		***
Mean	88,332.19	99,300.62	57,145.00	103,122.10	61,847.52
SD	192,378.30	220,274.80	54,600.61	233,721.50	66,260.18

Notes: SD = Standard deviation; Asterisk signals a significant difference between the aware and unaware, and the difference between the engaged and non-engaged, which was determined by Chi-square test for categorical variables or two-tailed *t* test for continuous variables; *** *p* < 0.001.

**Table 3:** OLS and binary logistic regression results of the influence of GDC on children’s BMI and overweight/obesity.

Variables	BMI		Overweight/Obesity	
	Model 1	Model 2	Model 3	Model 4
Aware of GDC (No)	−0.658 ***		−0.434 ***	
	(0.154)		(0.097)	
Engaged in GDC (No)		−0.492 ***		−0.380 ***
		(0.134)		(0.091)

Notes: OLS = ordinary least squares; GDC = Growth and Development Check; BMI = body mass index; All covariates in Table 2 were controlled. Robust standard errors in parentheses; *** *p* < 0.001.

**Table 4:** PSM results of the influence of GDC on children’s BMI and overweight/obesity.

Matching Method	BMI			Overweight/Obesity		
	ATT	S.E.	Z	ATT	S.E.	Z
Aware of GDC (No)						
Nearest neighbor matching	−0.693 **	0.237	−2.92	−0.101 ***	0.029	−3.43
Caliper matching	−0.669 ***	0.189	−3.54	−0.103 ***	0.023	−4.52
Kernel matching	−0.695 ***	0.175	−3.96	−0.108 ***	0.023	−4.69
Engaged in GDC (No)						
Nearest neighbor matching	−0.363 *	0.183	−1.99	−0.064 *	0.025	−2.54
Caliper matching	−0.344 *	0.142	−2.43	−0.062 ***	0.019	−3.28
Kernel matching	−0.355 *	0.142	−2.50	−0.063 ***	0.019	−3.31

Notes: PSM = propensity score matching; BMI = body mass index; ATT = average treatment effect on the treated; S.E. = standard error; Z = Z-value. The standard error was obtained by the bootstrap method (replicated 500 times); *** *p* < 0.001, ** *p* < 0.01, * *p* < 0.05.
